# A randomised controlled trial to assess the antithrombotic effects of aspirin in type 1 diabetes: role of dosing and glycaemic control

**DOI:** 10.1186/s12933-021-01427-y

**Published:** 2021-12-17

**Authors:** William A. E. Parker, Rebecca Sagar, Zeyad Kurdee, Fladia Hawkins, Khalid M. Naseem, Peter J. Grant, Robert F. Storey, Ramzi A. Ajjan

**Affiliations:** 1 Cardiovascular Research Unit, Department of Infection, Immunity and Cardiovascular Disease, University of Sheffield, Sheffield, UK; 2 Leeds Institute of Cardiovascular and Metabolic Medicine, University of Leeds, Leeds, UK; 3 Clinical Biochemistry Unit, Pathology Department, College of Medicine, King Saud University, Riyadh, Saudi Arabia

**Keywords:** Aspirin, Diabetes mellitus, Platelet inhibition, Fibrin

## Abstract

**Background:**

The enhanced thrombotic milieu in diabetes contributes to increased risk of vascular events. Aspirin, a key antiplatelet agent, has inconsistent effects on outcomes in diabetes and the best dosing regimen remains unclear. This work investigated effects of aspirin dose and interaction with glycaemia on both the cellular and protein components of thrombosis.

**Methods:**

A total of 48 participants with type 1 diabetes and 48 healthy controls were randomised to receive aspirin 75 or 300 mg once-daily (OD) in an open-label crossover study. Light transmittance aggregometry and fibrin clot studies were performed before and at the end of each treatment period.

**Results:**

Aspirin demonstrated reduced inhibition of collagen-induced platelet aggregation (PA) in participants with diabetes compared with controls, although the higher dose showed better efficacy. Higher aspirin dose facilitated clot lysis in controls but not individuals with diabetes. Collagen-induced PA correlated with glycaemic control, those in the top HbA1c tertile having a lesser inhibitory effect of aspirin. Threshold analysis suggested HbA1c levels of > 65 mmol/mol and > 70 mmol/mol were associated with poor aspirin response to 75 and 300 mg daily doses, respectively. Higher HbA1c was also associated with longer fibrin clot lysis time.

**Conclusions:**

Patients with diabetes respond differently to the antiplatelet and profibrinolytic effects of aspirin compared with controls. In particular, those with elevated HbA1c have reduced inhibition of PA with aspirin. Our findings indicate that reducing glucose levels improves the anti-thrombotic action of aspirin in diabetes, which may have future clinical implications.

**Trial registration:**

EudraCT, 2008-007875-26, https://www.clinicaltrialsregister.eu/ctr-search/search?query=2008-007875-26.

**Supplementary Information:**

The online version contains supplementary material available at 10.1186/s12933-021-01427-y.

## Introduction

Patients with diabetes mellitus, including type 1 (T1D) or type 2 (T2D), are at increased risk of atherothrombotic events, including acute coronary syndromes, thrombotic stroke and critical limb ischaemia [1, 2]. Platelet activation and fibrin network formation are key to the formation of intravascular obstructive thrombus in these conditions [3]. Aspirin (acetylsalicylic acid) is an irreversible inhibitor of cyclo-oxygenase (COX) enzymes. By inhibiting platelet COX-1, aspirin blocks the generation of the potent pro-aggregatory and vasoconstrictive factor thromboxane (TX) A_2_, whilst relatively sparing inhibition of anti-aggregatory and vasorelaxant prostacyclin, which may be synthesised in the endothelium by COX-1 or COX-2 depending on the setting [4]. As well as an antiplatelet effect, aspirin can acetylate fibrinogen, which affects fibrin network structure and facilitates fibrinolysis, a potential additional mechanism for the beneficial effects for aspirin following a cardiovascular event [5, 6].

Whilst there is good evidence for the use of aspirin in the secondary prevention of atherothrombotic events, the results of primary prevention trials in diabetes have led to controversy [7]. In the ASCEND trial (A Study of Cardiovascular Events in Diabetes), the most recent and largest study to date comprising 15,480 individuals with diabetes (94% with T2D) without pre-existing cardiovascular disease, aspirin therapy at a dose of 100 mg/day was associated with no net clinical benefit to justify its routine use in primary prevention [8].

It has been suggested that, in patients with diabetes, aspirin may be less efficacious than in people without diabetes, perhaps related to glycation of platelet proteins and/or clotting factors, making them less accessible for acetylation by the drug [9]. Similarly, accelerated platelet turnover in patients with T2D, remaining understudied in T1D, may be a further factor that reduces the efficacy of maintenance aspirin dosing [10].

One hypothetical strategy to mitigate any reduced efficacy of aspirin in those with diabetes may be to increase the dose. The current recommended dose of aspirin for cardiovascular prophylaxis is 75**–**100 mg once daily (OD) [11]. This is largely based on an analysis by the Antithrombotic Trialists’ Collaboration, including a population with only a 4% prevalence of diabetes (of any type), that suggested higher doses offered no additional benefit in reducing ischaemic events [12]. There are also concerns that higher doses of aspirin may counterproductively inhibit endothelial prostacyclin release and lead to greater risk of gastroduodenal ulceration [13].

We hypothesised that high levels of glycation, caused by high average glycaemia, impair protein acetylation in diabetes and attenuate the antithrombotic effects of aspirin. Therefore, the aim of our work was to compare the effects of two different clinically-available doses of aspirin in patients with diabetes versus healthy controls in a randomised-controlled crossover trial and study the antithrombotic response both at the cellular and protein arms of blood coagulation. Individuals with type 1 diabetes (T1D) with no advanced complications were recruited into this trial to enable the study of potential interactions between glycation and acetylation away from other confounding variables commonly found in those with type 2 diabetes.

## Methods

### Study population

A single-centre, hospital outpatient-based randomised-controlled crossover trial was performed in 48 participants with T1D and 48 healthy controls. In the T1D group, participants had to be aged 18–50 years with a diagnosis of T1D treated with insulin only, using reliable contraception and not receiving any other medication. The control group met the same criteria apart from the fact they did not have a diagnosis of diabetes and were recruited from local hospital and university staff. Those with current or prior antithrombotic treatment or an indication for it, significant co-morbidity, receiving any drug other than insulin, with a contraindication to aspirin or, in the control group, evidence of diabetes were excluded. Full exclusion criteria are provided in Additional file 1.

### Study treatments

**Fig. 1:**
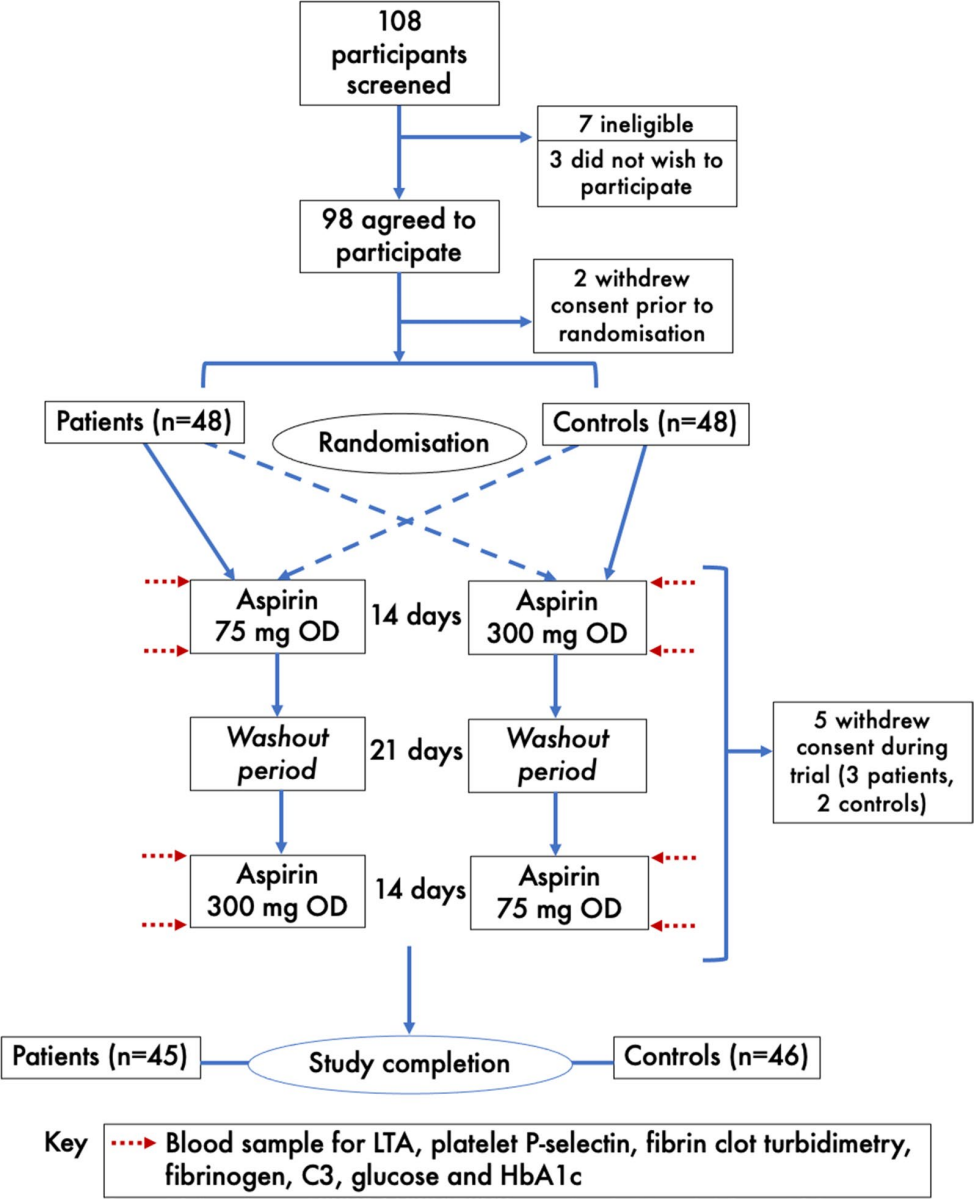
Design of the study. C3, complement component 3; HbA1c, glycated haemoglobin A1c; LTA, light transmittance aggregometry; OD, once daily

Participants were randomised in an unrestricted manner to one of two medication sequences in a 1:1 crossover design (Fig. 1). Randomisation was performed by a member of staff outside the research team who sealed and shuffled envelopes containing an equal number of allocations to each sequence. Members of the research team then opened the envelopes and assigned participants to the directed sequence, cross-checked by pharmacy colleagues at time of drug prescription. Each participant received, in a randomised order, 14 days of aspirin 75 mg OD and 14 days of 300 mg OD. The two periods were separated by a three-week washout period. Study medication was open-label and was obtained from local hospital supplies of generic dispersible aspirin. A treatment period length of 14 days was chosen to ensure steady state of aspirin’s effect and that there had been total platelet turnover during treatment [14], eliminating any chance of a carryover effect, whilst minimising drug exposure and maximising feasibility of the study. There were no significant changes to the study design after commencement.

### Platelet function testing

The principal antithrombotic effect of aspirin is to reduce platelet activation, thus impairing aggregation. To study this, light transmittance aggregometry (LTA) was performed on platelet-rich plasma, prepared by centrifugation of citrated blood at 200 g for 10 min at room temperature, using collagen (2 µg/mL) and arachidonic acid (AA, 1 mmol/L) as agonists and a PAP-4 aggregometer (Bio/Data Corporation, Horsham, PA, USA), as previously described [15]. Maximum platelet aggregation (maxPA) responses at 6 min after addition of agonist, adjusted for baseline, were recorded. Samples were assessed in duplicate, taking the mean value for analysis, and repeated if a discrepancy of >10% was observed between the readings.

### Fibrin clot turbidimetric analysis

As well as inhibiting platelets, aspirin may also have effects on the acellular arm of coagulation by modulating fibrin clot characteristics [5]. To assess the effect of aspirin dosing on fibrin clot dynamics, high-throughput turbidimetric analysis was performed as previously described and validated [16–20]. Briefly, lysis mix was added to citrated platelet-poor plasma samples before adding an activation mix that resulted in fibrin clot formation and subsequent lysis. Serial absorbance was measured using an automated plate reader during clot formation until lysis was achieved. Variables recorded were lag time, representing the period from the addition of clot activation mix to the start of clot formation (a measure of clotting tendency), final clot turbidity (maximum absorbance, a representation of fibre thickness and clot density), and lysis time (time from full clot formation to 50% lysis, a measure of fibrinolysis potential).

### Fibrinogen and complement C3

Fibrinogen levels, which can affect both clot formation and lysis, and can be associated with long-term cardiovascular risks in patients with diabetes [21], were determined by the clotting method of Clauss using a KC 10TM coagulometer (Henrich Amelung GmbH, Lemgo, Germany), as described elsewhere [22, 23]. Levels of complement C3, an inflammatory protein with anti-fibrinolytic properties, were determined, as previously described [18].

### Statistical analysis

All participants who completed the study were included in the analysis set. The primary endpoint of the study was the change in maxPA response to collagen from start to end of each treatment period compared between patients with T1D and controls for each aspirin regimen using unpaired t-tests. The secondary endpoint was maxPA response to collagen stratified by HbA1c level. maxPA responses AA; fibrin clot dynamics; and fibrinogen and C3 levels were exploratory endpoints, as were other analyses performed to gain further understanding of the effects seen. A primary aim of the study was also to estimate the prevalence of poor aspirin response, using an established definition of on-treatment PA response > 80% compared with off-treatment level when assessed using 2 µg/mL-collagen as an agonist during LTA [24]. Correlations between continuous variables were assessed using the Pearson method. GraphPad PRISM v7, RStudio v1.1.456 and SPSS Statistics v25 were used for analyses. Corrections for multiple tests were not made and therefore the results of secondary and exploratory analyses are considered to be hypothesis-generating only.

### Power calculation

Using previous data on platelet inhibition by aspirin treatment in patients with diabetes [20], a group size of 45 was deemed sufficient to detect a 10% difference in maxPA response to 2 µg/mL-collagen between groups with a power of 90% (at p = 0.05), based on the assumption that within-patient standard deviation of the response variable is 14%. The same number was enough to detect a 7% difference in clot final turbidity and 13% difference in lysis, given SD of the response variables at 10% and 19%, respectively (power of 90%, at p < 0.05) [25–27].

### Trial conduct

The trial protocol was approved by the National Health Service Research Ethics Service and the Medicines and Healthcare Products Regulatory Agency. The study was sponsored and monitored by the University of Leeds. Written informed consent was obtained from each participant prior to any study activities. The trial was conducted in accordance with Good Clinical Practice and the Declaration of Helsinki (1964).

## Results

Of 108 potential participants screened, 101 were found to be eligible. Ninety-eight agreed to participate but 2 withdrew consent before starting the study and 5 during the trial, leaving a total of 45 patients with T1D and 46 controls completing the study and therefore being included in analysis of the results (Fig. 1). Baseline characteristics are shown and compared in Table 1. There were no significant within-subject differences in any study endpoint between baseline values at the start of each of the two treatment periods (Additional file 1: Tables S1, S2). Recruitment and follow-up took place over a period of 22 months, and the trial closed only once the recruitment target had been met and all follow-up completed. No Good Clinical Practice-defined serious adverse reactions occurred during the trial.

**Table 1:** Baseline characteristics of participants completing the study

	Controls (n = 46)	Patients (n = 45)	p value
Sex (female/male)	20/26 (43.5%/56.5%)	19/26 (42.2%/57.8%)	> 0.99
Age (years)	24.4 ± 6.4	26.0 ± 6.8	0.23
History of smoking, n (%)	2 (4.3%)	9 (20.0%)	0.03
Blood pressure			
Systolic (mmHg)	116.8 ± 12.3	114.8 ± 11.2	0.42
Diastolic (mmHg)	76.3 ± 9.7	78.7 ± 8.7	0.21
Physical examination			
Height (m)	1.73 ± 0.1	1.73 ± 0.1	0.74
Weight (kg)	69.9 ± 11.3	72.9 ± 9.9	0.17
BMI (kg/m^2^)	23.2 ± 2.9	24.6 ± 3.5	0.04
Baseline blood tests			
HbA1c (mmol/mol)	35.2 ± 2.9	70.3 ± 17.7	< 0.0001
Sodium (mmol/L)	140.9 ± 1.9	139.8 ± 2.4	0.02
Potassium (mmol/L)	4.0 ± 0.3	4.1 ± 0.3	0.06
Creatinine (µmol/L)	80.9 ± 13.1	78.5 ± 20.7	0.50
Urea (mmol/L)	4.9 ± 1.2	5.3 ± 1.3	0.16
Bilirubin (µmol/L)	11.0 ± 6.5	11.9 ± 6.5	0.43
ALT (IU/L)	21.2 ± 8.4	19.3 ± 8.4	0.24
ALP (IU/L)	166.0 ± 48.5	203.5 ± 70.6	0.004
Albumin (g/L)	46.0 ± 2.4	44.7 ± 2.6	0.02
Total cholesterol (mmol/L)	4.2 ± 0.9	4.4 ± 0.7	0.23
LDL (mmol/L)	2.4 ± 0.8	2.5 ± 0.5	0.43
HDL (mmol/L)	1.5 ± 0.4	1.6 ± 0.4	0.24
Triglycerides (mmol/L)	0.96 ± 0.31	0.90 ± 0.35	0.34
FT_4_ (pmol/L)	14.8 ± 2.2	15.0 ± 1.7	0.70
TSH (mIU/L)	2.4 ± 1.0	2.3 ± 1.3	0.84
Haemoglobin (g/dL)	14.2 ± 1.2	14.4 ± 1.0	0.22
Leukocyte count (×10^9^/L)	6.1 ± 1.5	6.4 ±1.7	0.43
Platelet count (×10^9^/L)	266 ± 53	279 ± 67	0.29
Time since diabetes diagnosis (months)	–	124.8 ± 101.8	–
Family history			
Ischaemic heart disease	16 (34.8%)	9 (20.0%)	0.16
Autoimmunity	11(23.9%)	16 (35.6%)	0.26

Where appropriate, controls and patients were compared using Fisher’s exact test for proportions and two-tailed unpaired t-tests for continuous variables. Values for continuous variables are expressed as mean ± SD. ALP, alkaline phosphatase; ALT, alanine transferase; BMI, body mass index; FT_4_, free thyroxine; Hb, haemoglobin; HDL, high-density lipoprotein; LDL, low-density lipoprotein; TSH, thyroid stimulating hormone

### Platelet aggregation responses

**Fig. 2:**
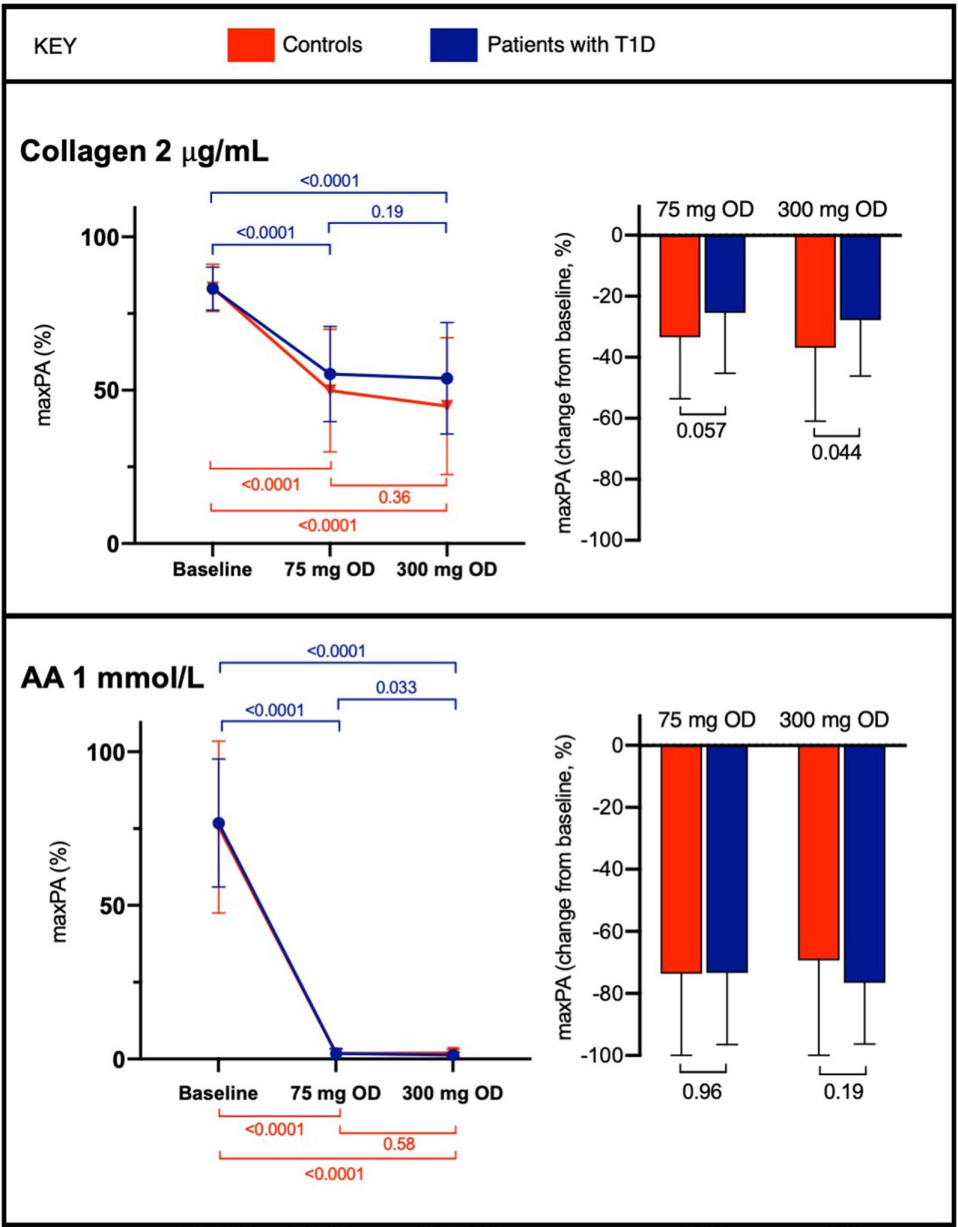
Maximum platelet aggregation (maxPA) responses to collagen and arachidonic acid (AA) in controls and patients with type 1 diabetes (T1D) at baseline and when receiving aspirin 75 mg or 300 mg once daily (OD). Bars represent mean ± SD. P values are shown for within-participant comparisons (left) and between groups (right)

There were no significant differences in baseline maxPA responses between controls and patients (Additional file 1: Table S1, Fig. 2).

In controls and patients, both doses of aspirin significantly reduced maxPA responses to collagen 2 µg/mL (Fig. 2). When receiving either dose, the reduction from baseline was less in patients than controls, with the difference being statistically significant when receiving aspirin 300 mg once daily but not 75 mg once daily. maxPA responses to 1 mmol/L-AA were significantly reduced in both groups by either aspirin dose, and there were no differences in the changes from baseline between patients and controls (Fig. 2). In patients, but not controls, maxPA responses to 1 mmol/L-AA, but not collagen 2 µg/mL, were significantly lower when receiving aspirin 300 mg once daily when compared to 75 mg OD (Additional file 1: Table S1, Fig. 2), though the mean difference was only – 0.51% (95% confidence interval – 0.98 to 0.04). When defined as on-treatment 2 µg/mL-collagen-induced PA response > 80% compared with off-treatment level, poor aspirin response was seen in 24% when receiving aspirin 75 mg OD and 22% when receiving 300 mg OD.

### Fibrin clot characteristics

**Fig. 3:**
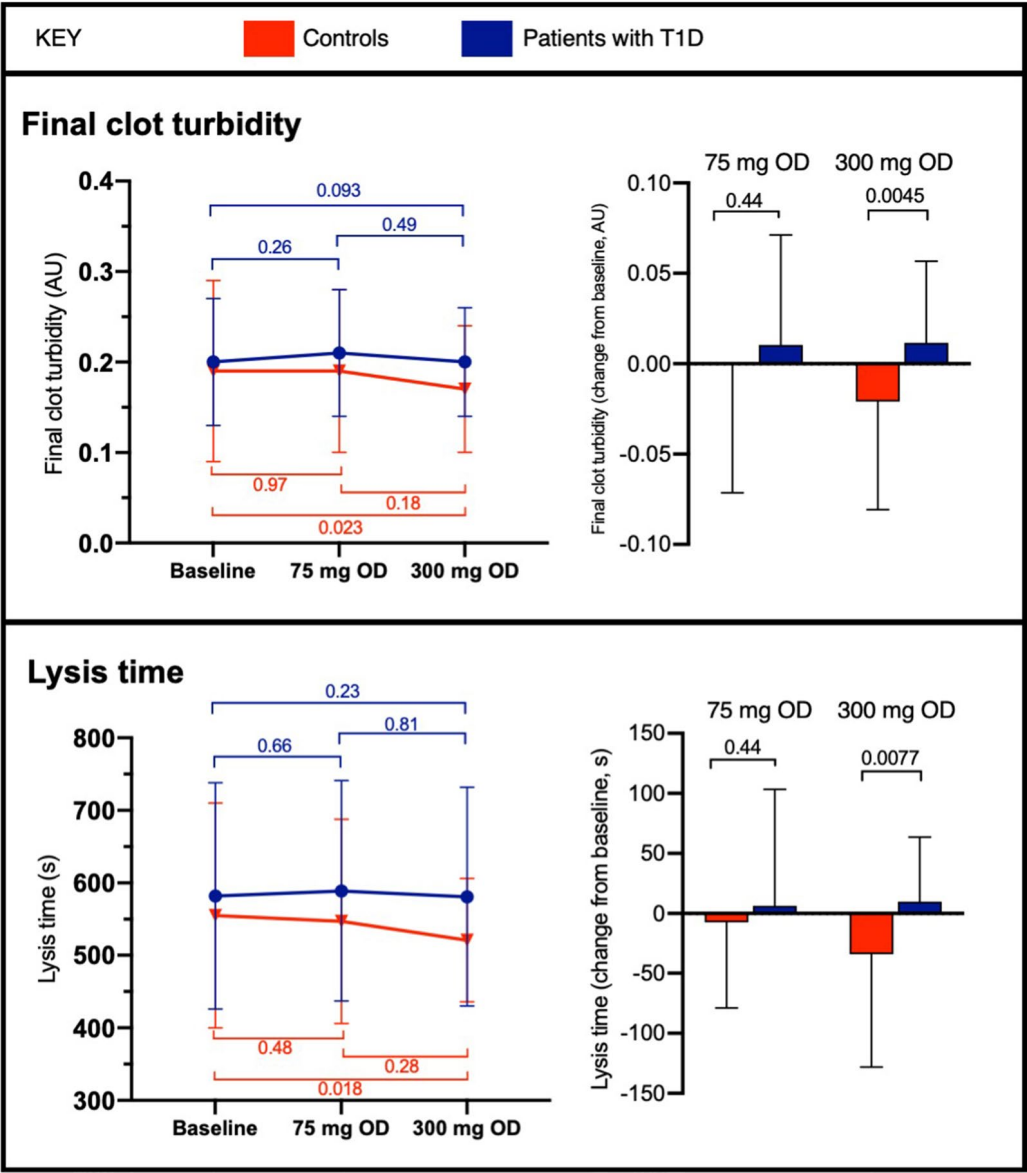
Fibrin clot final turbidity and lysis time in controls and patients with type 1 diabetes (T1D) at baseline and when receiving aspirin 75 mg or 300 mg once daily (OD). Bars represent mean ± SD. P values are shown for within-participant comparisons (left) and between groups (right)

At baseline, there were no significant differences in fibrin clot characteristics when compared between controls and patients (Additional file 1: Table S2, Fig. 3). Similarly, there were no significant differences in the changes from baseline between the groups when receiving aspirin 75 mg OD (Fig. 3). However, when receiving aspirin 300 mg OD, change in final clot turbidity and lysis time was significantly more pronounced in controls than patients, the latter group displaying no effect. Neither aspirin nor diabetes status had any significant effect on clot lag time (Additional file 1: Table S2).

### Plasma levels of fibrinogen and complement C3

**Fig. 4:**
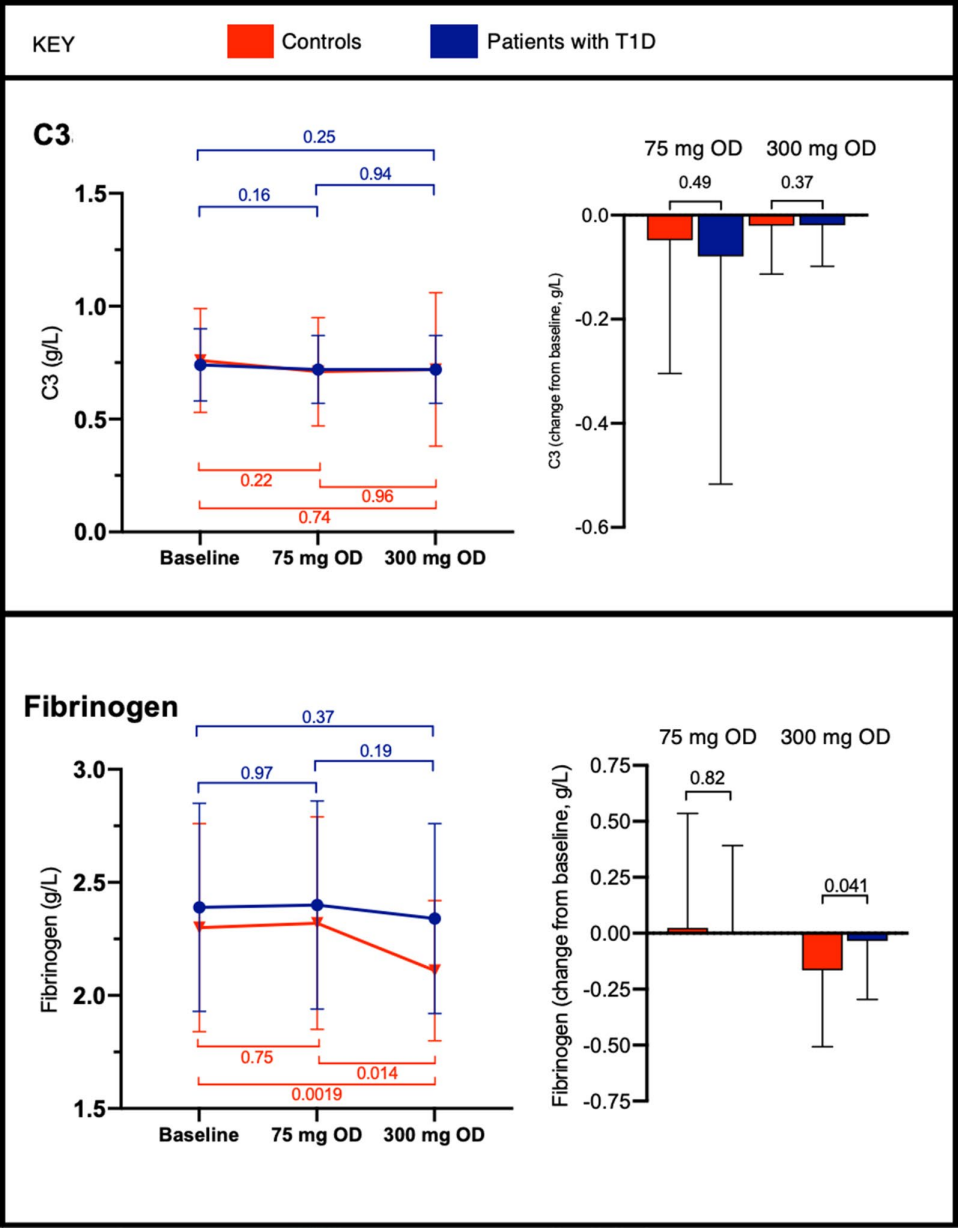
Plasma fibrinogen and complement component 3 (C3) levels in controls and patients with type 1 diabetes (T1D) at baseline and when receiving aspirin 75 mg or 300 mg once daily (OD). Bars represent mean ± SD. P values are shown for within-participant comparisons (left) and between groups (right)

Fibrinogen and C3 levels can affect fibrin clot characteristics [28], and therefore plasma levels of these proteins were investigated. No difference in fibrinogen or C3 levels were detected at baseline, comparing patients and controls (Additional file 1: Table S2). However, higher-dose aspirin resulted in reduced fibrinogen levels in controls, but not patients (Fig. 4). C3 levels were not affected by aspirin administration (Fig. 4).

### Correlation with glycation and glycaemic status in patients with T1DM

**Fig. 5:**
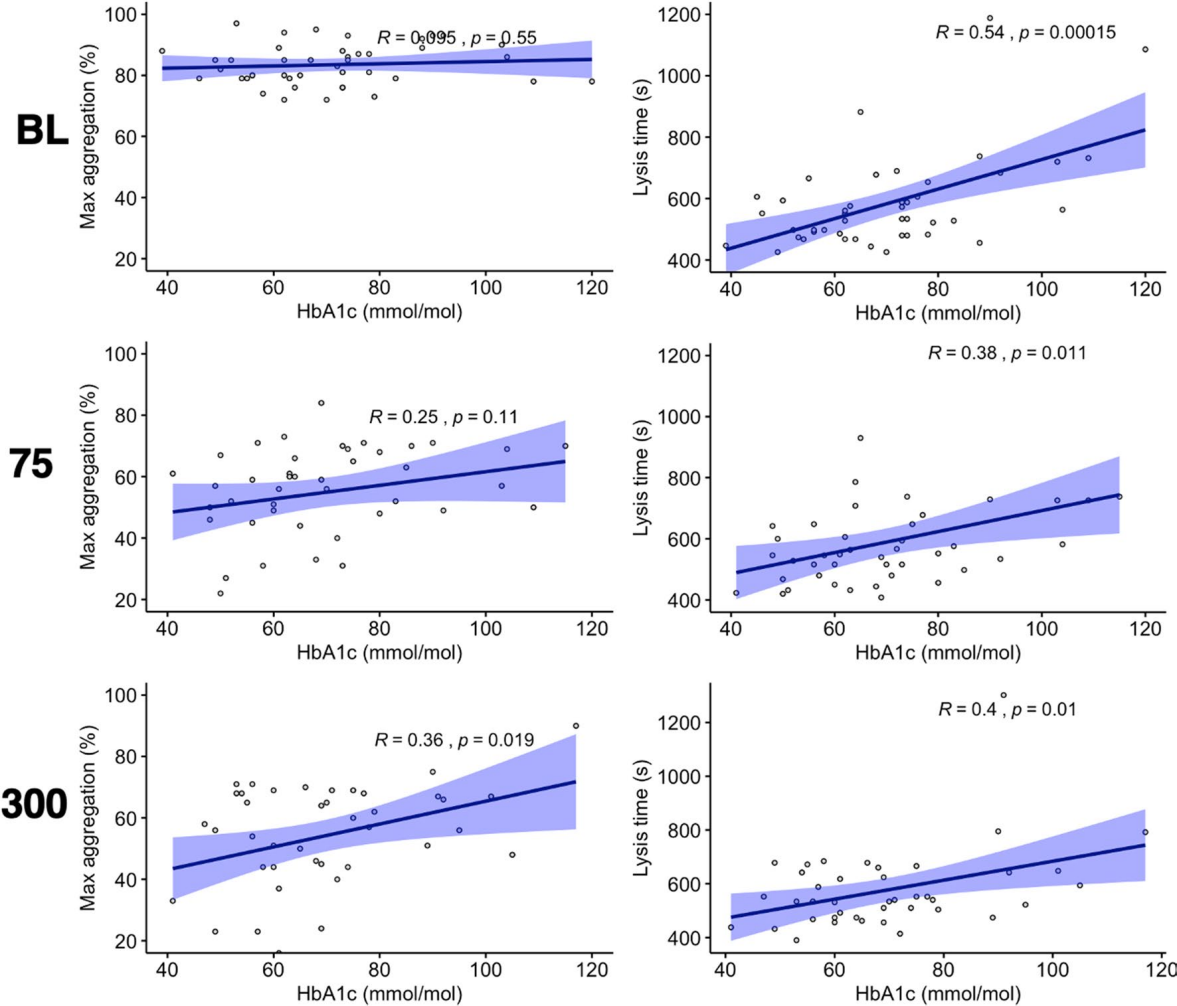
Correlation between glycated haemoglobin (Hb)A1c and either maximum platelet aggregation response to collagen 2 µg/mL (left) or fibrin clot lysis time (right) at baseline (BL, top) and during treatment with aspirin 75 mg once daily (75, middle) or 300 mg once daily (300, bottom) in patients with type 1 diabetes. Solid blue line represents line of regression and shaded area represents 95% CI

Levels of glycated haemoglobin A1c (HbA1c) had no significant correlation with maxPA responses at baseline or when receiving aspirin 75 mg OD (Table 2; Fig. 5). When receiving 300 mg OD, there was a significant, positive correlation between HbA1c and maxPA responses to collagen (Table 2; Fig. 5). Further analysis, however, suggested no significant correlation between HbAlc and difference in collagen-induced maxPA responses when receiving aspirin 75 mg OD vs. 300 mg OD (Additional file 1: Fig. S1).

**Table 2:** Correlation between glycaemic control, measured as haemoglobin (Hb)A1c, plasma glucose levels and study endpoints in type 1 diabetes patients at baseline and when receiving aspirin 75 mg or 300 mg once daily (OD)

Parameter	Correlation with HbA1c						Correlation with plasma glucose					
	Baseline		75 mg OD		300 mg OD		Baseline		75 mg OD		300 mg OD	
	R	p	R	p	R	p	R	p	R	p	R	p
Platelet aggregation												
AA 1 mmol/L	0.24	0.11	0.17	0.28	0.13	0.41	0.058	0.71	0.12	0.47	0.054	0.73
Collagen 2 µg/mL	0.095	0.55	0.25	0.11	**0.36**	**0.019**	− 0.13	0.42	0.032	0.84	0.096	0.54
Collagen 16 µg/mL	0.076	0.62	− 0.015	0.93	0.23	0.14	0.15	0.33	− 0.06	0.71	0.068	0.67
Fibrin clot turbidimetry												
Lag time (s)	0.17	0.27	0.0032	0.98	− 0.11	0.48	0.36	0.019	0.044	0.79	0.046	0.77
Final clot turbidity (AU)	**0.57**	**< 0.0001**	**0.54**	**0.00021**	**0.45**	**0.0033**	0.2	0.19	0.31	0.05	0.24	0.12
Lysis time (s)	**0.54**	**0.00015**	**0.38**	**0.011**	**0.4**	**0.01**	0.046	0.77	0.059	0.71	0.21	0.17
Fibrinogen and C3												
Fibrinogen (g/L)	**0.48**	**0.00099**	**0.32**	**0.034**	**0.48**	**0.0017**	**0.35**	**0.022**	0.09	0.57	0.18	0.24
C3 (g/L)	0.24	0.12	0.21	0.18	0.094	0.56	0.011	0.94	0.097	0.55	− 0.24	0.13

Data generated using the Pearson method. Values in bold indicate those associated with a p value < 0.05

Regarding fibrin clot dynamics, at baseline and when receiving aspirin 75 or 300 mg OD, HbA1c positively and significantly correlated with final clot turbidity and lysis time (Table 2; Fig. 5). There was no significant correlation with lag time at any timepoint.

Levels of HbA1c positively and significantly correlated with plasma fibrinogen at baseline and when receiving either aspirin dose (Table 2). No correlation between C3 levels and HbA1c was observed at baseline or during treatment with aspirin.

At baseline, there was a positive and significant correlation between plasma glucose and fibrinogen levels as well as lag time (Table 2). During aspirin treatment at either dose, there was no significant correlation between plasma glucose and any study endpoint.

### Threshold analysis of the relationship between HbA1c and poor aspirin response

**Fig. 6:**
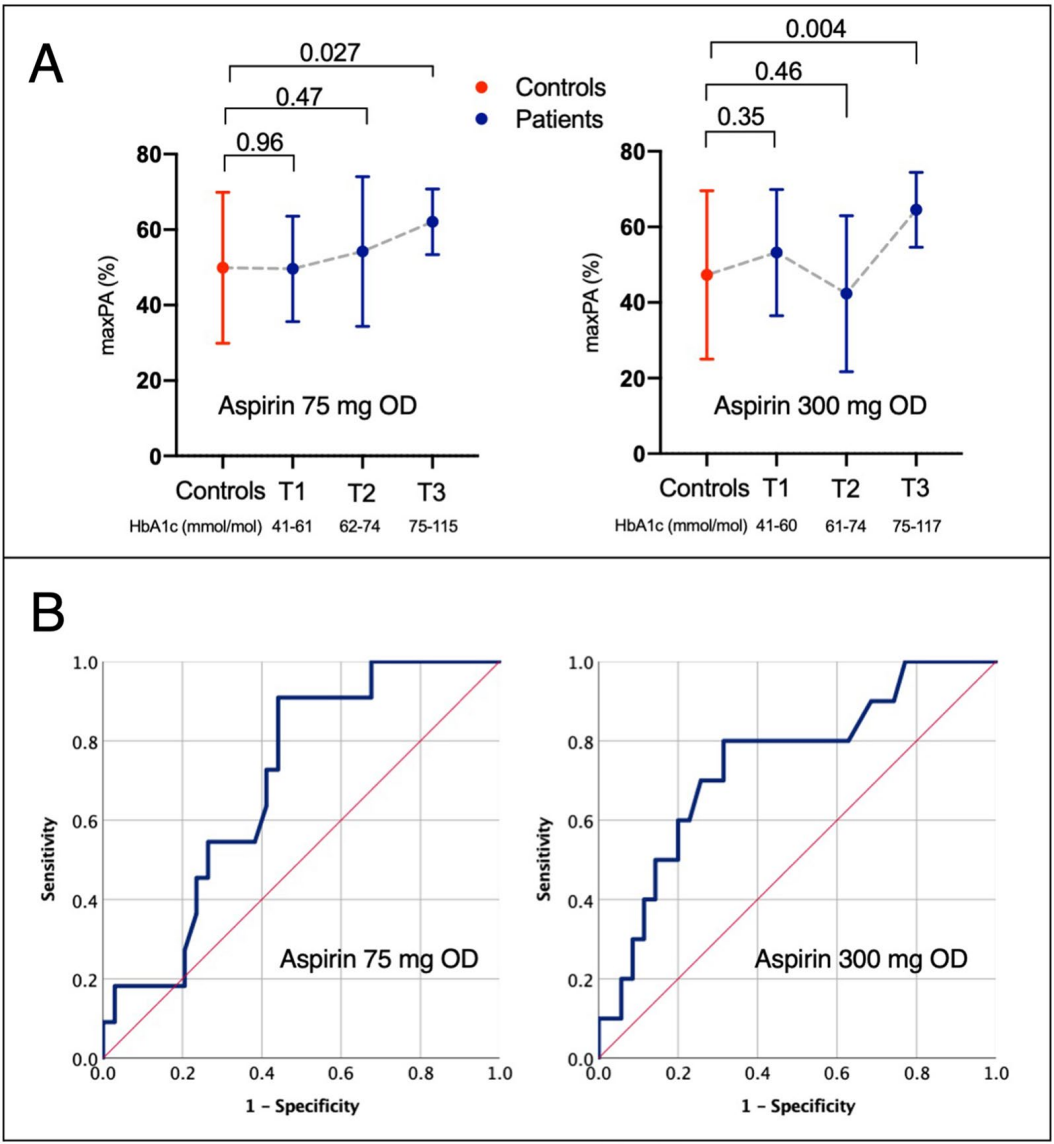
Relationship between haemoglobin (Hb)A1c and collagen-induced maximum platelet aggregation (maxPA) responses when receiving aspirin 75 mg or 300 mg once daily (OD), presented by HbA1c tertile (T) (panel **A**, p values shown for controls vs. tertile) and as receiver operating characteristic curves for predicting poor response to aspirin (panel **B**)

To further explore the influence of glycation status on response to aspirin in patients with diabetes, tertiles of HbA1c were investigated in relation to collagen-induced PA responses. Whether receiving aspirin 75 mg OD or 300 mg OD, those in the highest tertile had less inhibitory effect of aspirin (Fig. 6).

The ability of HbA1c to predict poor response to aspirin treatment was also assessed using a binary logistic regression model. Poor response to aspirin has been defined as an on-treatment PA response > 80% compared with off-treatment level when assessed using 2 µg/mL-collagen as an agonist during LTA [24]. Simple logistic regression was used to identify factors associated with poor aspirin response when receiving aspirin 75 mg OD or 300 mg OD (Additional file 1: Table S3). Testing threshold values of HbA1c ascending in 5 mmol/mol steps suggested that an HbA1c of > 65 mmol/mol was significantly associated with poor aspirin response when receiving aspirin 75 mg OD (exp[B] = 5.70, p = 0.042) and > 70 mmol/mol when receiving 300 mg OD (exp[B] = 6.77, p = 0.027). Receiver operating characteristic curves were constructed to further explore the ability of HbA1c level to predict poor aspirin response (Fig. 6). These demonstrated fair predictive value when receiving either 75 mg OD (area under curve [AUC] = 0.70) or 300 mg OD (AUC = 0.74). The cut-off of > 65 mmol/mol when receiving 75 mg OD offered a sensitivity of 82% and specificity of 56%, whereas > 70 mmol/mol when receiving 300 mg OD offered 80% sensitivity and 69% specificity.

Using a previously described approach [29], including variables with a univariate p value of <0.15 in a multivariate logistic regression model for each regimen, suggested that the threshold of > 70 mmol/mol when receiving 300 mg OD was an independent predictor of poor aspirin response (exp[B] = 5.87, p = 0.045). However, the threshold of > 65 mmol/L when receiving 75 mg OD did not reach a significant statistical level to demonstrate this (exp[B] = 3.77, p = 0.16, Additional file 1: Table S4–S5).

## Discussion

It is generally believed that a daily aspirin dose of 75–100 mg is sufficient to maximally inhibit platelet COX-1 activity [13]. However, this has been based on findings from cohorts with a low incidence of diabetes, a condition that may hypothetically reduce the effectiveness of aspirin through glycation of its target proteins [9]. Our data suggest that aspirin was not as effective as reducing PA in patients with T1D compared to controls, particularly when receiving 300 mg OD. Whilst increasing aspirin dose from 75 mg OD to 300 mg OD offered significantly greater inhibition of AA-induced in patients with T1D, the difference was small and collagen-induced responses were similar. Combined with the fact that higher-dose aspirin may hypothetically increase counteractive effects on endothelial prostacyclin release, gastroduodenal integrity and haemostasis, which were not assessed in this study, it seems unlikely that this would translate into a clinical benefit in this patient group [30]. This is supported by recent data on clinical outcomes from Aspirin Dosing: A Patient-Centric Trial Assessing Benefits and Long-Term Effectiveness (ADAPTABLE), in which the aspirin regimens 81 mg OD and 325 mg OD were compared in 15,076 patients with established atherosclerotic cardiovascular disease [31]. After a median of 26 months, there was no significant difference in the rates of a composite primary endpoint of all-cause death, hospitalisation for myocardial infarction or hospitalisation for stroke (7.28% [81 mg] vs. 7.51% [325 mg]; hazard ratio 1.02; 95% confidence interval 0.91 to 1.14; p = 0.75). Furthermore, this finding appeared replicated in the subgroup (n = 5676) with diabetes (HR 0.99 [0.84 to 1.17]).

Fibrin clot dynamics may also be an important determinant of thrombotic risk. For example, lysis time is an independent predictor of cardiovascular risk after an acute coronary syndrome event, including in those with diabetes [6]. Fibrin clot lysis time was shortened and clot maximum absorbance reduced following 300 mg OD aspirin in controls, consistent with previous work using aspirin 150 mg OD [5], while 75 mg OD had no effect. In contrast, neither dose of aspirin affected lysis or clot maximum turbidity in those with T1D; therefore aspirin failed to modulate fibrin clot properties in our patient population (at least using 75 and 300 mg daily doses). Interestingly, fibrinogen levels were reduced by 300 mg aspirin in controls but not diabetes patients, providing one mechanism for the observed changes in fibrin clot parameters. This may be due to an anti-inflammatory effect for the higher aspirin dose, which appears to only affect individuals without diabetes. Work on the fibrin network in T1D is limited with a previous study showing that only higher dose aspirin (320 mg OD) affects fibrin clot permeability in these individuals, which was more pronounced in those with poor glycaemic control, but fibrin clot lysis was not studied [32]. However, we did not investigate clot permeability given that the role of this fibrin parameter in predisposition to cardiovascular events is unknown.

There was evidence of correlation between platelet function or fibrin clot dynamics during aspirin treatment and HbA1c but not plasma glucose. Furthermore, we determined that poor response to aspirin was particularly associated with an HbA1c level > 65–70 mmol/mol, those with a lower level having similar responses to controls. This supports the hypothesis that aspirin may be blocked from acetylating target proteins if these are glycated, rather than merely in the presence of hyperglycaemia. Our findings demonstrate that increasing aspirin dose from 75 mg OD to 300 mg OD does not overcome poor response in patients with high glucose levels, and, above all, our findings support the importance of good glycaemic control in reducing thrombotic risk. Our data suggest that avoiding an overly elevated level HbA1c may improve the antiplatelet response to aspirin and confer more advantageous fibrin clot dynamics. Further work is required to explore this hypothesis.

Beyond a dose increase, other hypothetical strategies for improving the strength and reliability of aspirin’s antithrombotic effect in patients with diabetes might include adding a second antiplatelet agent such as a P2Y_12_ inhibitor (dual antiplatelet therapy, DAPT). Though there is evidence that diabetes patients with established coronary artery disease may gain benefits of reduced ischaemic risk from long-term DAPT compared to aspirin alone [33, 34], there is currently no evidence for use of P2Y_12_ inhibitors for primary prevention of cardiovascular events in any population, including in those with diabetes. An alternative similarly unexplored regimen for primary prevention in patients with diabetes might be to combine aspirin with a low-dose anticoagulant drug such as the non-vitamin K antagonist oral anticoagulant, rivaroxaban [35]. As well as aspirin dose, increased frequency of dosing beyond OD may hypothetically improve aspirin exposure and counter high platelet turnover in this group, though whether this would overcome poor aspirin response in patients with very high HbA1c has not been well studied [36]. Intensifying antithrombotic therapy typically attracts a penalty of increased bleeding risk that would need to be carefully balanced to prove net clinical benefit in any trial in this population. Other approaches such as P2Y_12_ inhibitor monotherapy or very-low-dose twice-daily aspirin-based regimens may plausibly help to better achieve an optimum balance of benefits and risks [37, 38]. Further study of alternative treatment options to standard doses of OD aspirin for primary prevention in patients with diabetes is needed.

There are clear strengths to this study that should be highlighted. First, we recruited young T1D individuals, thus limiting confounders, which allowed the assessment of the role of glycaemia in response to aspirin treatment. Second, the work studied both the cellular and protein arms of coagulation, thereby giving a comprehensive assessment of thrombosis potential. Finally, withdrawal rate was low with over 90% randomised participants completing the study. A limitation of this study is that levels of TXA_2_ and prostacyclin release were not directly assessed. The study did not assess clinical outcomes of ischaemic or bleeding events, but is valuable in providing mechanistic insights into the findings of trials that have done so. We cannot be sure that our findings are applicable to patients with T2D, but given hyperglycaemia and hyperglycation are common to both T1D and T2D, it appears rational to expect this to be the case, though study specifically in T2D would be needed to definitively confirm it.

## Conclusions

Patients with diabetes have an impaired response to aspirin compared to healthy controls with reduced platelet inhibition and absent profibrinolytic activity. Increasing the dose of aspirin in individuals with diabetes had little effect on the cellular and protein arms of coagulation, and the reduced response to this agent appears to be related to inadequate diabetes control. Therefore, improving glycaemia may be necessary to optimise the anti-thrombotic effect of aspirin in diabetes.

## Supplementary Information

**Additional file 1.** Additional details of study design and further data analyses.

## Data Availability

All data generated or analysed during this study are included in this published article and its Additional file 1.

## Abbreviations

AA: Arachidonic acid
ALP: Alkaline phosphatase
ALT: Alanine transferase
ASCEND: A Study of Cardiovascular Events in Diabetes
AUC: Area under curve
C3: Complement component 3
COX: Cyclo-oxygenase
FT4: Free thyroxine
HbA1c: Haemoglobin A1c
HDL: High-density lipoprotein
LDL: Low-density lipoprotein
LTA: Light transmittance aggregometry
