# Supplementary material for: A randomised controlled trial to assess the antithrombotic effects of aspirin in type 1 diabetes: role of dosing and glycaemic control
Source: Cardiovasc Diabetol. 2021 Dec 17;20:238. doi: 10.1186/s12933-021-01427-y (PMC8684134; doi:10.1186/s12933-021-01427-y)
Supplement: Supplementary file 1 — Additional file 1. Additional details of study design and further data analyses. [file 12933_2021_1427_MOESM1_ESM.docx]

**Additional file 1**

**Study exclusion criteria assessed prior to randomisation**

(a) Prior treatment with aspirin, clopidogrel or warfarin. Regular previous or

current treatment with non-steroidal inflammatory drugs (NSAIDs), current treatment

with any medication other than insulin

(b) any history of acute coronary syndrome or stroke, deep vein thrombosis,

pulmonary embolism, previous or current upper gastrointestinal pathology,

malignancy or coagulation disorders. Any individual found to have abnormal liver function (measured by alanine transferase>3 fold upper limit of normal) or abnormal thyroid function will be excluded and offered further investigation

(c) Control subjects will be excluded if found to have impaired glucose

tolerance or diabetes measured according to current guidelines

(d) Not currently using contraception

(e) Pregnant, or lactating, women will not be included in the study. In the unlikely

event of pregnancy during the study, the individual will be immediately withdrawn

(f) Gout or a history of gout

(g) Hypersensitivity to aspirin or other NSAIDs

**Table S1** Results of light transmittance aggregometry and exploratory comparisons at baseline and during treatment with aspirin 75 mg or 300 mg once daily (OD). AA, arachidonic acid.

|  | Maximum aggregation (%) (Mean ± SD) | | | | | | | | Comparisons (p values) | | | | |
| --- | --- | --- | --- | --- | --- | --- | --- | --- | --- | --- | --- | --- | --- |
|  | Controls | | | | Patients | | | | Controls | Patients | Controls vs. Patients | | |
| Agonist | Baseline | 75 mg OD | Baseline | 300 mg OD | Baseline | 75 mg OD | Baseline | 300 mg OD | Baseline vs. Baseline | Baseline vs. Baseline | Baseline vs. Baseline* | 75 mg OD vs 75 mg OD | 300 mg OD vs 300 mg OD |
| AA 1 mmol/L | 75.5 ± 28.0 | 1.8 ± 1.5 | 71.3 ± 31.5 | 2.0 ± 1.2 | 76.8 ± 20.8 | 1.8 ± 1.5 | 79.6 ± 16.3 | 1.3 ± 0.9 | 0.37 | 0.48 | 0.79 | 0.95 | 0.0054 |
| Collagen 2 μg/mL | 83.4 ± 7.7 | 49.9 ± 20.0 | 83.7 ± 6.7 | 44.8 ± 22.3 | 83.1 ± 7.0 | 55.3 ± 15.5 | 82.3 ± 6.6 | 53.9 ± 18.2 | 0.83 | 0.51 | 0.87 | 0.16 | 0.1 |

*Analysis performed using baseline data from start of 75 mg OD period

**Table S2** Results of fibrin clot turbidimetry, fibrinogen and complement component 3 (C3) assays, and exploratory comparisons. OD, once daily.

|  | Maximum aggregation (%) (Mean ± SD) | | | | | | | | Comparisons (p values) | | | | |
| --- | --- | --- | --- | --- | --- | --- | --- | --- | --- | --- | --- | --- | --- |
|  | Controls | | | | Patients | | | | Controls | Patients | Controls vs. Patients | | |
| Parameter | Baseline | 75 mg OD | Baseline | 300 mg OD | Baseline | 75 mg OD | Baseline | 300 mg OD | Baseline vs. Baseline | Baseline vs. Baseline | Baseline vs. Baseline* | 75 mg OD vs 75 mg OD | 300 mg OD vs 300 mg OD |
| Lag time (s) | 744.7 ± 131.2 | 743 ±161 | 760.8 ± 136.2 | 775 ± 153 | 770.5 ±156.4 | 734 ± 125 | 789.6 ± 195.2 | 766 ± 170 | 0.56 | 0.63 | 0.4 | 0.76 | 0.79 |
| Final clot turbidity (AU) | 0.19 ± 0.10 | 0.19 ± 0.09 | 0.19 ± 0.08 | 0.17 ± 0.07 | 0.20 ± 0.07 | 0.21 ± 0.07 | 0.19 ± 0.07 | 0.20 ± 0.06 | 0.9 | 0.45 | 0.46 | 0.17 | 0.0083 |
| Lysis time (s) | 555 ± 155 | 547 ± 141 | 555 ± 133 | 521 ± 85 | 582 ± 156 | 589 ± 152 | 571 ± 148 | 581 ± 151 | 0.99 | 0.72 | 0.4 | 0.18 | 0.021 |
| Fibrinogen (g/L) | 2.30 ± 0.46 | 2.32 ± 0.47 | 2.25 ± 0.33 | 2.11 ± 0.31 | 2.39 ± 0.46 | 2.40 ± 0.49 | 2.38 ± 0.49 | 2.34 ± 0.42 | 0.57 | 0.88 | 0.33 | 0.47 | 0.0039 |
| C3 (g/L) | 0.76 ± 0.23 | 0.71 ± 0.24 | 0.81 ± 0.34 | 0.72 ± 0.36 | 0.74 ± 0.16 | 0.72 ± 0.15 | 0.74 ± 0.16 | 0.72 ± 0.15 | 0.37 | 0.99 | 0.66 | 0.81 | 0.95 |

*Analysis performed using baseline data from start of 75 mg OD period

**Table S3** Factors associated with poor aspirin response determined by univariate analysis using simple logistic regression. ACR, albumin creatinine ratio, ALP, alkaline phosphatase; ALT, alanine transferase; BMI, body mass index; eGFR, estimated glomerular filtration rate; Hb, haemoglobin; T_4_, thyroxine; TSH, thyroid stimulating hormone.

| **Aspirin Regimen** | 75 mg OD | | | | | 300 mg OD | | | | |
| --- | --- | --- | --- | --- | --- | --- | --- | --- | --- | --- |
| **Factor** | **B** | **SE** | **Wald** | **p value** | **Exp(B)** | **B** | **SE** | **Wald** | **p value** | **Exp(B)** |
| Age (years) | -0.187 | 0.088 | 4.524 | 0.033 | 0.829 | -0.056 | 0.062 | 0.822 | 0.364 | 0.945 |
| Sex | -0.323 | 0.716 | 0.204 | 0.652 | 0.724 | -0.118 | 0.73 | 0.026 | 0.872 | 0.889 |
| BMI (kg/m^2^) | -0.103 | 0.106 | 0.934 | 0.334 | 0.902 | 0.198 | 0.114 | 2.979 | 0.084 | 1.218 |
| Weight (kg) | -0.07 | 0.04 | 3.031 | 0.082 | 0.932 | 0.001 | 0.037 | 0.001 | 0.975 | 1.001 |
| Time since diabetes diagnosis (months) | -0.007 | 0.005 | 2.739 | 0.098 | 0.993 | 0 | 0.004 | 0.007 | 0.932 | 1 |
| Smoking status | 0.242 | 0.527 | 0.21 | 0.647 | 1.273 | 0.037 | 0.572 | 0.004 | 0.949 | 1.038 |
| Platelet count (x10^9^/L) | 0 | 0.005 | 0.003 | 0.954 | 1 | 0.004 | 0.005 | 0.505 | 0.477 | 1.004 |
| Glucose (mmol/L) | 0.059 | 0.083 | 0.502 | 0.479 | 1.061 | 0.061 | 0.078 | 0.621 | 0.431 | 1.063 |
| TSH (mIU/L) | 0.249 | 0.248 | 1.007 | 0.316 | 1.282 | -0.066 | 0.294 | 0.05 | 0.823 | 0.936 |
| ALT (IU/L) | -0.012 | 0.05 | 0.058 | 0.809 | 0.988 | -0.007 | 0.051 | 0.018 | 0.893 | 0.993 |
| ALP (IU/L) | 0.006 | 0.005 | 1.796 | 0.18 | 1.006 | 0.002 | 0.005 | 0.235 | 0.628 | 1.002 |
| HbA1c (mmol/mol) | 0.036 | 0.02 | 3.158 | 0.076 | 1.037 | 0.048 | 0.022 | 4.791 | 0.029 | 1.049 |
| Total cholesterol (mmol/L) | -0.079 | 0.514 | 0.023 | 0.878 | 0.924 | 0.312 | 0.519 | 0.362 | 0.548 | 1.366 |
| ACR (mg/mmol) | 0.087 | 0.076 | 1.311 | 0.252 | 1.091 | -0.793 | 0.822 | 0.93 | 0.335 | 0.452 |
| Haemoglobin (g/dL) | 0.014 | 0.355 | 0.001 | 0.969 | 1.014 | 0.377 | 0.409 | 0.848 | 0.357 | 1.457 |
| Leukocyte count (x10^9^/L) | -0.121 | 0.225 | 0.291 | 0.59 | 0.886 | 0.04 | 0.209 | 0.036 | 0.849 | 1.04 |
| Testosterone (nmol/L) | -0.008 | 0.039 | 0.037 | 0.847 | 0.992 | 0.002 | 0.04 | 0.002 | 0.967 | 1.002 |
| Vitamin D (ng/mL) | -0.008 | 0.039 | 0.037 | 0.847 | 0.992 | 0.002 | 0.04 | 0.002 | 0.967 | 1.002 |
| eGFR (ml/min) | 0.006 | 0.053 | 0.013 | 0.909 | 1.006 | 0.016 | 0.058 | 0.073 | 0.787 | 1.016 |
| Albumin (g/L) | 0.077 | 0.137 | 0.316 | 0.574 | 1.08 | -0.002 | 0.14 | 0 | 0.988 | 0.998 |
| Free T_4_ (pmol/L) | 0.006 | 0.053 | 0.013 | 0.909 | 1.006 | 0.016 | 0.058 | 0.073 | 0.787 | 1.016 |
| HbA1c >50 mmol/mol | 20.269 | 16408.704 | 0 | 0.999 | 634650696 | 20.071 | 20096.481 | 0 | 0.999 | 521120876 |
| HbA1c >55 mmol/mol | 20.382 | 13397.656 | 0 | 0.999 | 710808920 | 20.173 | 15191.516 | 0 | 0.999 | 576955303 |
| HbA1c >60 mmol/mol | 1.696 | 1.109 | 2.342 | 0.126 | 5.455 | 1.417 | 1.115 | 1.615 | 0.204 | 4.125 |
| HbA1c >65 mmol/mol | 1.74 | 0.855 | 4.147 | 0.042 | 5.7 | 1.214 | 0.86 | 1.993 | 0.158 | 3.368 |
| HbA1c >70 mmol/mol | - | - | - | - | - | 1.912 | 0.865 | 4.893 | 0.027 | 6.769 |

**Table S4** Multivariate analysis of poor aspirin response during treatment with aspirin 75 mg once daily, including factors with p<0.15 on univariate analysis, using binary logistic regression. BMI, body mass index; Hb, haemoglobin.

| **Factor** | **B** | **SE** | **Wald** | **p value** | **Exp(B)** |
| --- | --- | --- | --- | --- | --- |
| BMI (kg/m^2^) | -0.024 | 0.127 | 0.036 | 0.849 | 0.976 |
| Time since diabetes diagnosis (months) | -0.004 | 0.006 | 0.500 | 0.480 | 0.996 |
| Age (years) | -0.102 | 0.098 | 1.092 | 0.296 | 0.903 |
| HbA1c >65 mmol/mol | 1.326 | 0.952 | 1.942 | 0.163 | 3.766 |

**Table S5** Multivariate analysis of poor aspirin response during treatment with aspirin 300 mg once daily, including factors with p<0.15 on univariate analysis, using binary logistic regression. BMI, body mass index; Hb, haemoglobin.

| **Factor** | **B** | **SE** | **Wald** | **p value** | **Exp(B)** |
| --- | --- | --- | --- | --- | --- |
| BMI (kg/m^2^) | 0.161 | 0.118 | 1.884 | 0.170 | 1.175 |
| HbA1c >70 mmol/mol | 1.770 | 0.882 | 4.025 | 0.045 | 5.872 |

**Figure S1** Correlation between haemoglobin A1c (HbAlc) and individual difference in collagen-induced maximum platelet aggregation (maxPA) responses between the regimens (calculated as maxPA when receiving 300 mg OD – maxPA when receiving 75 mg OD), analysed using the Pearson test.


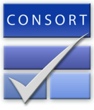
CONSORT 2010 checklist of information to include when reporting a randomised trial

| Section/Topic | Item No | Checklist item | Reported on page No* |
| --- | --- | --- | --- |
| Title and abstract | | | |
|  | 1a | Identification as a randomised trial in the title | 1 |
|  | 1b | Structured summary of trial design, methods, results, and conclusions (for specific guidance see CONSORT for abstracts) | 2 |
| Introduction | | | |
| Background and objectives | 2a | Scientific background and explanation of rationale | 3 |
|  | 2b | Specific objectives or hypotheses | 4 |
| Methods | | | |
| Trial design | 3a | Description of trial design (such as parallel, factorial) including allocation ratio | 4, Figure 1 |
|  | 3b | Important changes to methods after trial commencement (such as eligibility criteria), with reasons | 5 |
| Participants | 4a | Eligibility criteria for participants | 4/5, Appendix |
|  | 4b | Settings and locations where the data were collected | 4 |
| Interventions | 5 | The interventions for each group with sufficient details to allow replication, including how and when they were actually administered | 5 |
| Outcomes | 6a | Completely defined pre-specified primary and secondary outcome measures, including how and when they were assessed | 6/7 |
|  | 6b | Any changes to trial outcomes after the trial commenced, with reasons | N/A |
| Sample size | 7a | How sample size was determined | 7 |
|  | 7b | When applicable, explanation of any interim analyses and stopping guidelines | N/A |
| Randomisation: |  |  |  |
| Sequence generation | 8a | Method used to generate the random allocation sequence | 5 |
|  | 8b | Type of randomisation; details of any restriction (such as blocking and block size) | 5 |
| Allocation concealment mechanism | 9 | Mechanism used to implement the random allocation sequence (such as sequentially numbered containers), describing any steps taken to conceal the sequence until interventions were assigned | 5 |
| Implementation | 10 | Who generated the random allocation sequence, who enrolled participants, and who assigned participants to interventions | 5 |
| Blinding | 11a | If done, who was blinded after assignment to interventions (for example, participants, care providers, those assessing outcomes) and how | N/A |
|  | 11b | If relevant, description of the similarity of interventions | N/A |
| Statistical methods | 12a | Statistical methods used to compare groups for primary and secondary outcomes | 6/7 |
|  | 12b | Methods for additional analyses, such as subgroup analyses and adjusted analyses | 6/7 |
| Results | | | |
| Participant flow (a diagram is strongly recommended) | 13a | For each group, the numbers of participants who were randomly assigned, received intended treatment, and were analysed for the primary outcome | Fig 1 |
|  | 13b | For each group, losses and exclusions after randomisation, together with reasons | Fig 1 |
| Recruitment | 14a | Dates defining the periods of recruitment and follow-up | 8 |
|  | 14b | Why the trial ended or was stopped | 8 |
| Baseline data | 15 | A table showing baseline demographic and clinical characteristics for each group | Table 1 |
| Numbers analysed | 16 | For each group, number of participants (denominator) included in each analysis and whether the analysis was by original assigned groups | Table 1 |
| Outcomes and estimation | 17a | For each primary and secondary outcome, results for each group, and the estimated effect size and its precision (such as 95% confidence interval) | 7-11 |
|  | 17b | For binary outcomes, presentation of both absolute and relative effect sizes is recommended | N/A |
| Ancillary analyses | 18 | Results of any other analyses performed, including subgroup analyses and adjusted analyses, distinguishing pre-specified from exploratory | 7-11 |
| Harms | 19 | All important harms or unintended effects in each group (for specific guidance see CONSORT for harms) | 8 |
| Discussion | | | |
| Limitations | 20 | Trial limitations, addressing sources of potential bias, imprecision, and, if relevant, multiplicity of analyses | 13 |
| Generalisability | 21 | Generalisability (external validity, applicability) of the trial findings | 13 |
| Interpretation | 22 | Interpretation consistent with results, balancing benefits and harms, and considering other relevant evidence | 11/12 |
| Other information | | |  |
| Registration | 23 | Registration number and name of trial registry | 2 |
| Protocol | 24 | Where the full trial protocol can be accessed, if available | N/A |
| Funding | 25 | Sources of funding and other support (such as supply of drugs), role of funders | 16 |

*page numbers refer to submitted main manuscript file

N/A, not applicable
